# Low-Temperature (≤500 °C) Complementary Schottky Source/Drain FinFETs for 3D Sequential Integration

**DOI:** 10.3390/nano12071218

**Published:** 2022-04-05

**Authors:** Shujuan Mao, Jianfeng Gao, Xiaobin He, Weibing Liu, Jinbiao Liu, Guilei Wang, Na Zhou, Yanna Luo, Lei Cao, Ran Zhang, Haochen Liu, Xun Li, Yongliang Li, Zhenhua Wu, Junfeng Li, Jun Luo, Chao Zhao, Wenwu Wang, Huaxiang Yin

**Affiliations:** 1 Institute of Microelectronics, Chinese Academy of Sciences, Beijing 100029, China; maoshujuan@ime.ac.cn (S.M.); gaojianfeng@ime.ac.cn (J.G.); hexiaobin@ime.ac.cn (X.H.); liuweibing@ime.ac.cn (W.L.); liujinbiao@ime.ac.cn (J.L.); zhouna@ime.ac.cn (N.Z.); luoyanna@ime.ac.cn (Y.L.); caolei@ime.ac.cn (L.C.); zhangran@ime.ac.cn (R.Z.); liuhaochen@ime.ac.cn (H.L.); lixun@ime.ac.cn (X.L.); liyongliang@ime.ac.cn (Y.L.); wuzhenhua@ime.ac.cn (Z.W.); lijunfeng@ime.ac.cn (J.L.); luojun@ime.ac.cn (J.L.); wangwenwu@ime.ac.cn (W.W.); 2 Beijing Superstring Academy of Memory Technology, Beijing 100176, China; guilei.wang@bjsamt.org.cn (G.W.); chao.zhao@bjsamt.org.cn (C.Z.); 3 School of Microelectronics, University of Chinese Academy of Sciences, Beijing 100049, China

**Keywords:** 3D sequential integration, low thermal budget, Schottky S/D FinFETs, inverter

## Abstract

In this work, low-temperature Schottky source/drain (S/D) MOSFETs are investigated as the top-tier devices for 3D sequential integration. Complementary Schottky S/D FinFETs are successfully fabricated with a maximum processing temperature of 500 °C. Through source/drain extension (SDE) engineering, competitive driving capability and switching properties are achieved in comparison to the conventional devices fabricated with a standard high-temperature (≥1000 °C) process flow. Schottky S/D PMOS exhibits an ON-state current (*I*_ON_) of 76.07 μA/μm and ON-state to OFF-state current ratio (*I*_ON_/*I*_OFF_) of 7 × 10^5^, and those for NMOS are 48.57 μA/μm and 1 × 10^6^. The CMOS inverter shows a voltage gain of 18V/V, a noise margin for high (*NM*_H_) of 0.17 V and for low (*NM*_L_) of 0.43 V, with power consumption less than 0.9 μW at *V*_DD_ of 0.8 V. Full functionality of CMOS ring oscillators (RO) are further demonstrated.

## 1. Introduction

The technology of 2D planar scaling is now facing major limitations, and in order to extend the semiconductor roadmap, 3D sequential integration, which consists of stacking transistors on top of each other, has been envisioned [1,2]. As its name suggests, transistor layers are processed sequentially, i.e., the top tier is processed and stacked above the already fabricated bottom tier in 3D sequential integration. This technology can enhance circuit density and functionality without the requirement of further reduction in device dimensions. To maintain the integrity of what is below, namely the bottom devices, interconnections and bonding interface, the thermal budget for top-tier fabrication is required to be no more than 550 °C [3,4,5].

However, source/drain (S/D) activation is typically performed by spike annealing at high temperature (≥1000 °C). Decreasing thermal budget will impair CMOS device performance. Much work has been undertaken to circumvent the thermal budget limitation. For instance, nanosecond laser annealing (NLA) [6] and solid-phase epitaxial regrowth (SPER) [7] were used to activate S/D as the alternatives to high-temperature spike annealing and low-temperature materials, such as poly-Si [8,9], Ge [10,11], III-V [12,13] and transparent amorphous oxide [14,15] were implemented to replace monocrystalline Si as the channel of top-tier devices. Particularly interesting is the exploration of junctionless MOSFETs as the top-tier devices with the elimination of S/D activation [16,17]. Even though impressive device performances have been achieved with such approaches, there remain several issues. NLA and SPER often incur high process cost and low throughput, and low-temperature materials are not fully compatible with current Si technology, leading to a risk of poor yield at a large scale. Additionally, junctionless devices need an extra-high-temperature (≥1000 °C) annealing to activate the channel before the top silicon layer transfer, which is likely to induce mobility degradation and threshold voltage (*V*_TH_) variation. Hence, low-temperature devices based on Si technology may be further developed.

Schottky S/D MOSFETs, using metal silicide to replace doped silicon as S/D [18], hold an inherent superiority in process thermal budget over the conventional and junctionless devices, with no need of a standard high-temperature annealing to activate S/D and channel. Therefore, in this work, low-temperature complementary Schottky S/D FinFETs are processed at a temperature as low as 500 °C, and the electrical characteristics are investigated to evaluate the feasibility of being used as the top-tier logic devices in 3D sequential technology. To our knowledge, previous investigations on Schottky S/D devices have been mostly focused on short-channel effects, with the fabrication thermal budget never lower than 600 °C [19,20,21,22,23,24]. This is the first demonstration of complementary S/D FinFETs with all process steps below 500 °C toward 3D sequential integration.

## 2. Device Fabrication

**Figure 1:**
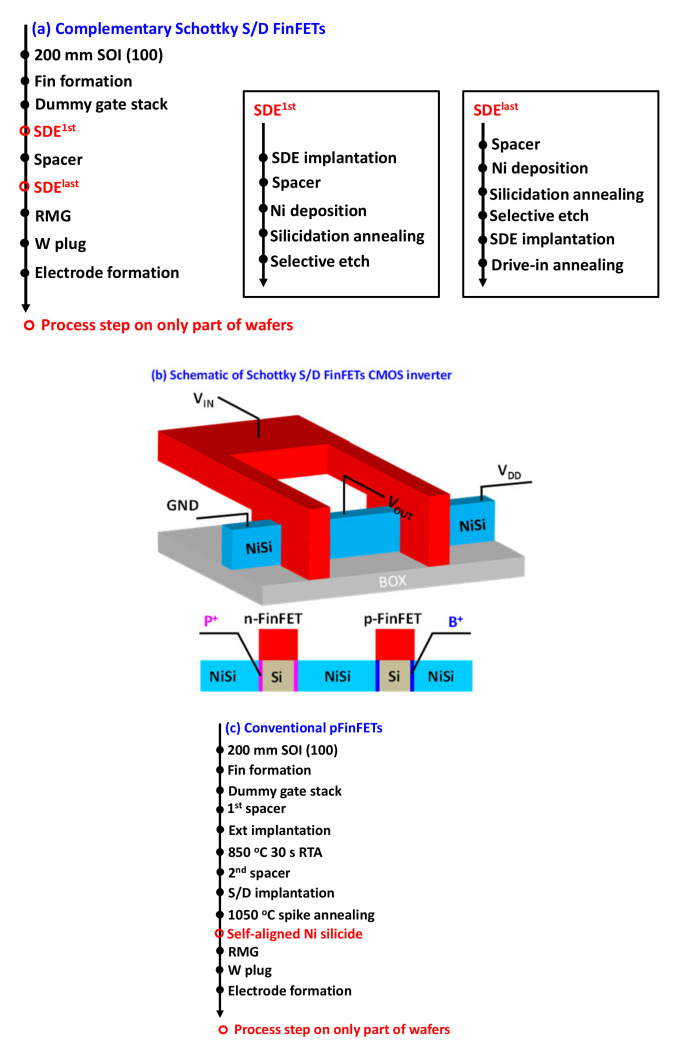
(**a**) Process flow of Schottky S/D FinFETs in this work, (**b**) Schematic layout and cross section of the complementary Schottky S/D FinFETs inverter, (**c**) Standard high-temperature process flow of conventional device.

The process flow of Schottky S/D FinFETs is summarized in Figure 1a. SOI wafers measuring 200 mm with top Si of 40 nm and BOX of 145 nm were used as the starting materials to mimic the bonded substrate of top-tier devices. The replacement metal gate (RMG) process was adopted, and all process steps were set below the typical thermal budget of 550 °C for compatibility with 3D sequential integration [3,4,5]. According to the principle of Schottky S/D MOSFETs [18], the electrical property is primarily determined by the Schottky junction barrier between S/D and channel. In order to realize high performance, a doped source/drain extension (SDE) to lower the Schottky junction barrier, illustrated in Figure 1b, was first explored for pFinFETs by two methods, i.e., SDE first (SDE^1st^) and SDE last (SDE^last^). In the SDE^1st^ scheme, SDE implantation was performed before the spacer and followed by silicidation annealing, and SDE was formed by dopant segregation at the silicide/Si interface during silicidation. In contrast, SDE implantation was performed after silicide formation in the SDE^last^ scheme, and an additional rapid thermal annealing (RTA), also named drive-in annealing, was used to drive the dopant to segregate at the silicide/Si interface, forming SDE. An amount of 3 nm Ni was deposited by sputtering, and B 1.5 keV 2 × 10^15^ cm^−^^2^ was implemented for SDE implantation in both schemes. A split, shown in Table 1, was further performed to investigate the impact of the SDE process thermal budget on device performance. Afterwards, the process flow of complementary Schottky S/D FinFETs was developed with optimal SDE engineering. Gate stacks of HfO_2_/TiN/TaN and HfO_2_/TiAl/TiN were separately applied to pFinFETs and nFinFETs for *V*_TH_ adjustment. The fabrication was completed with tungsten contact plug and Al metallization. Conventional pFinFETs, with and without silicide, were also fabricated with a standard high-temperature (≥1000 °C) process flow (Figure 1c) for comparison. It is worth noting that 8-inch industrial equipment was used for the fabrication in our experiment, and the process uniformity of within wafer, wafer-to-wafer and lot-to-lot was controllable and reproducible. Current-voltage measurements (112 measurement sites for each wafer) were performed using a HP4156 parameter analyser.

## 3. Results and Discussion

### 3.1. SDE Engineering

**Figure 2:**
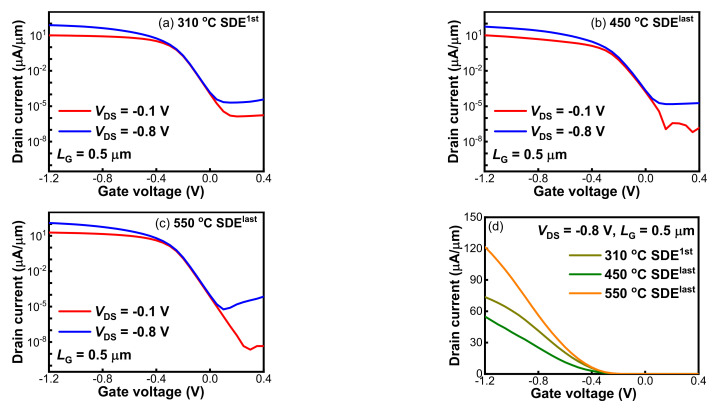
*I*_DS_-*V*_GS_ characteristics of the Schottky S/D pFinFETs with (**a**) 310 °C SDE^1st^, (**b**) 450 °C SDE^last^ and (**c**) 550 °C SDE^last^ processes; (**d**) Comparison of *I*_DS_ at *V*_DS_ = −0.8 V between the three.

To develop the optimal SDE process, SDE engineering, shown in Table 1, was performed on pFinFETs. The *I*_DS_-*V*_GS_ characteristics of the Schottky S/D pFinFETs with three SDE processes are noted in Figure 2a–c. Decent switching properties are demonstrated for all three, with ON-state to OFF-state current ratios (*I*_ON_/*I*_OFF_) of 5 orders, and excellent swings (SSs) of around 61 mV/decade obtained for physical gate length (*L*_G_) of 500 nm. As compared in Figure 2d, the top *I*_ON_ was achieved with 550 °C SDE^last^, followed by 310 °C SDE^1st^ and then 450 °C SDE^last^, with the values of 55.49 μA/μm, 40.78 μA/μm and 25.23 μA/μm at *V*_DS_ = −0.8 V; whereas larger *I*_OFF_ of 0.39 nA/μm and 0.21 nA/μm were resolved for 310 °C SDE^1st^ and 450 °C SDE^last^ with respect to that of 0.08 nA/μm for 550 °C SDE^last^. Further, the *V*_TH_ defined as *V*_GS_ corresponding to *I*_DS_ = 10^−7^ A/μm were −0.13 V, −0.16 V and −0.16 V for the devices with 310 °C SDE^1st^, 450 °C SDE^last^ and 550 °C SDE^last^ processes, respectively. A reduction of around 30 mV in *V*_TH_ was demonstrated for 310 °C SDE^1st^.

**Figure 3:**
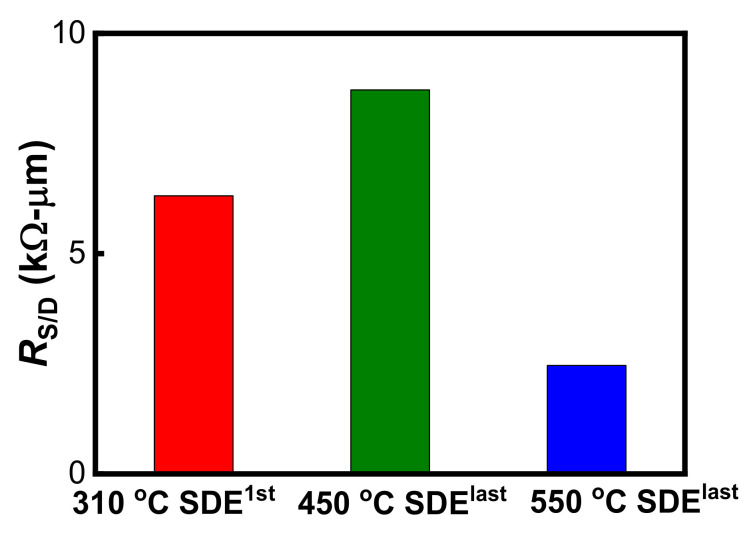
*R*_S/D_ at *V*_DS_ = −0.1 V of the Schottky S/D pFinFETs with different SDE processes.

**Figure 4:**
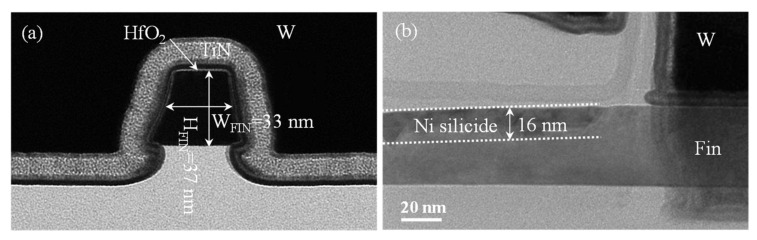
Cross-sectional transmission electron microscope (XTEM) images of the Schottky S/D pFinFETs (**a**) across and (**b**) along fins with gate stack covering, fabricated with 550 °C SED^last^ process.

**Figure 5:**
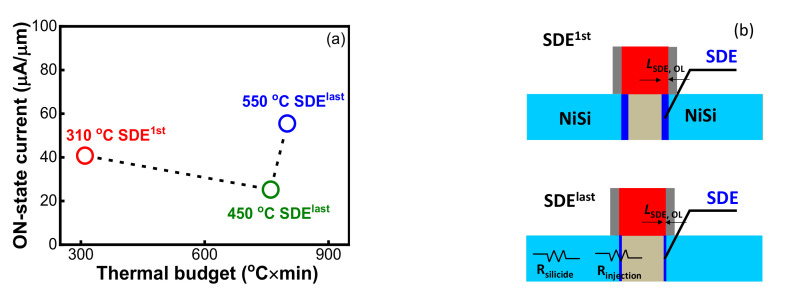
(**a**) *I*_ON_ dependence on SDE process thermal budget for Schottky S/D pFinFETs, (**b**) Schematic of gate-to-SDE overlap (*L*_SDE, OL_) and *R*_S/D_ component for the devices with SDE^1st^ and SDE^last^ processes.

Figure 3 shows the S/D series resistance (*R*_S/D_) extracted by fitting ON-state resistance (*R*_ON_) vs. 1/(*V*_GS_-*V*_TH_) at high gate bias in linear mode [25]. The *R*_S/D_ measurements at *V*_GS_ = −0.8 V were about 6.31 kΩ/μm, 8.71 kΩ/μm and 2.46 kΩ/μm for the devices with 310 °C SDE^1st^, 450 °C SDE^last^ and 550 °C SDE^last^ processes, respectively. Such high *R*_S/D_ values are ascribed to the nano-fins scheme and to insufficiently silicided S/D, as uncovered in Figure 4. Due to the ultrathin Ni of 3 nm, about 16 nm of silicide was formed, and yet around 24 nm silicon remained unsilicided. Figure 5a further shows the *I*_ON_ dependence on the SDE process thermal budget, which is involved with the annealing steps in Table 1. It should be noted that the heating-up and cooling-down periods of an annealing process are ignored when defining a thermal budget. It was revealed that lowering the SDE process thermal budget degraded *I*_ON_ in the SDE^last^ scheme; it seems that SDE^1st^ prevails over SDE^last^ in driving capability at a lower thermal budget level.

Correlating the electrical results with SDE engineering, two main findings can be made. First, SDE^1st^ holds the advantage in *I*_ON_ at a lower thermal budget level, which results from the larger gate-to-SDE overlap (*L*_SDE, OL_), by performing SDE implantation before the spacer, as illustrated in Figure 5b. The reduced *V*_TH_ of −0.13 V, larger *I*_OFF_ of 0.39 nA/μm and lower *R*_S/D_ of 6.31 kΩ/μm for 310 °C SDE^1st^, with reference to 450 °C SDE^last^, justify this point, which was performed with the same silicidation annealing. Second, in the SDE^last^ scheme, increasing the thermal budget will lower *R*_S/D,_ and thus improve *I*_ON_. The lowered *R*_S/D_ is probably attributable to the reductions in silicide resistance and injection resistance (Figure 5b). It is known that an Ni/Si solid state reaction forms high-resistance Ni_2_Si at 250–400 °C and low-resistance NiSi at 400–700 °C [26]. With the raising of silicidation annealing from 310 °C to 500 °C for 450 °C SDE^last^ and 550 °C SDE^last^ (Table 1), the sheet resistance of Ni silicide decreases from around 457 Ω to 123 Ω, measured with a four-point probe system. Additionally, it has been demonstrated that increasing drive-in annealing will boost dopant segregation at the silicide/Si interface, leading to an enhanced Schottky junction barrier lowering [27,28,29]. Since the injection resistance is proportional to the Schottky junction barrier, its reduction can be expected for 550 °C SDE^last^ with respect to 450 °C SDE^last^, with drive-in annealing at 550 °C for the former and 450 °C for the later. One may argue that the mobility could differ with SDE process thermal budget, affecting device performance. Since no channel doping was performed for Schottky devices and all samples were tested at 300 K, it is supposed that the mobility was almost the same and the difference in *I*_DS_-*V*_GS_ characteristics in Figure 2 was primarily caused by *R*_S/D_ and *V*_TH_.

### 3.2. Low-Temperature Schottky S/D FinFETs vs. Conventional High-Temperature FinFETs

**Figure 6:**
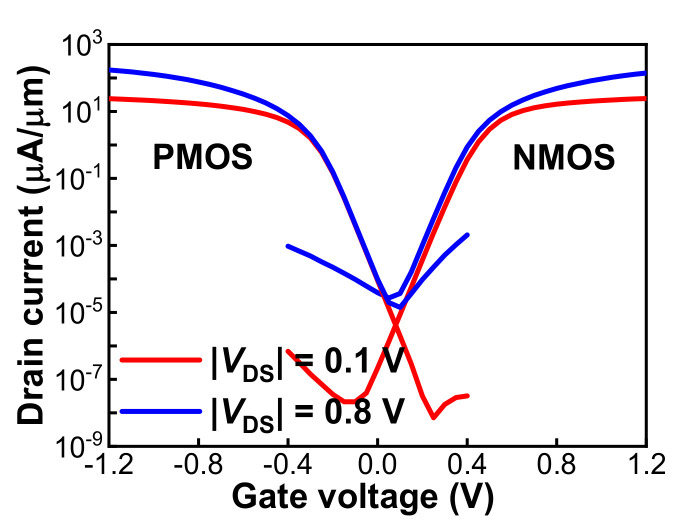
*I*_DS_-*V*_GS_ characteristics of low-temperature complementary Schottky S/D FinFETs with *L*_G_ = 500 nm.

In accordance with the findings in Section 3.1, the fabrication of complementary Schottky S/D FinFETs with optimal SDE engineering was developed toward 3D sequential integration. The thickness of Ni was increased from 3 nm to 6 nm and 5% Pt. was added, and a two-step RTA (310 °C 60 s + selective etch + 500 °C 10 s) method was explored for silicide formation so as to avoid abnormal Ni diffusion [30,31]. Meanwhile, the drive-in annealing was further reduced from 550 °C 60 s to 500 °C 60 s for better compatibility with 3D sequential integration. Figure 6 shows the *I*_DS_-*V*_GS_ characteristics of the complementary Schottky S/D FinFETs with *L*_G_ = 500 nm. The *I*_ON_ and *I*_ON_/*I*_OFF_ ratios for pFinFETs were 76.07 μA/μm and 7 × 10^5^, and those for nFinFETs were 48.57 and 1 × 10^6^ μA/μm, at *V*_DS_ = ±0.8 V. The corresponding *V*_TH_ values were around −0.16 V and 0.3 V. Clearly, with S/D fully silicided as confirmed by XTEM images (not shown), an improvement in *I*_ON_ was achieved for pFinFETs due to the reduced *R*_S/D_.

**Figure 7:**
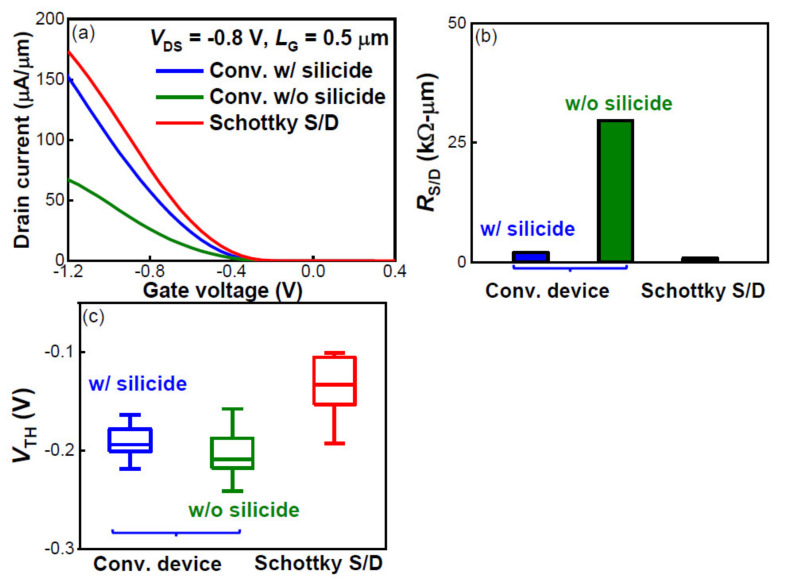
Comparison of (**a**) *I*_DS_ at *V*_DS_ = −0.8 V, (**b**) *R*_S/D_ and (**c**) *V*_TH_ between low-temperature Schottky S/D device and conventional (conv.) high-temperature device.

A comparison was made between low-temperature Schottky S/D pFinFETs and conventional pFinFETs fabricated with a standard high-temperature (≥ 1000 °C) process flow (Figure 1c). As shown in Figure 7a, with silicide formation, the *I*_ON_ of conventional pFinFETs is significantly improved. Since no shift of *V*_TH_ was evidenced with silicide (Figure 7c), the *I*_ON_ improvement is primarily attributable to the decrease in *R*_S/D_ from 29 kΩ/μm to 2 kΩ/μm (Figure 7b). The low-temperature Schottky S/D pFinFETs exhibited higher *I*_ON_ than the conventional high-temperature devices, whether they were with (w/) or without (w/o) silicide, owing to the lower *R*_S/D_ (Figure 7b) and *V*_TH_ (Figure 7c). As compared to the conventional device, the Schottky S/D device using silicide as S/D holds an inherent advantage in *R*_S/D,_ and its fabrication is fully compatible with current Si technology; competitive performance can be obtained with a 500 °C drive-in annealing to form SDE; moreover, no annealing at ≥1000 °C is needed to activate the channel with respect to a junctionless device [16,17]. Hence, it is indeed feasible to adopt Schottky S/D FinFETs as the top-tier devices in 3D sequential technology.

### 3.3. Inverter Characterization

**Figure 8:**
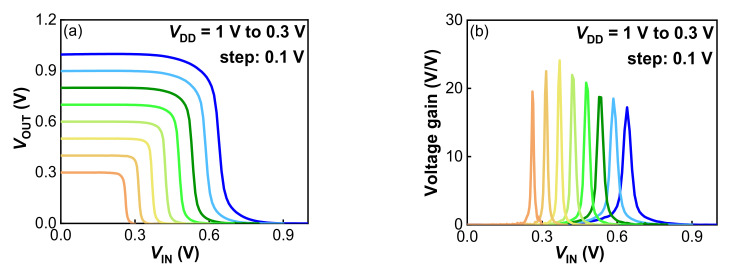
(**a**) Inverter VTC at *V*_DD_ ranging from 1 V down to 0.3 V, (**b**) Corresponding voltage gains.

**Figure 9:**
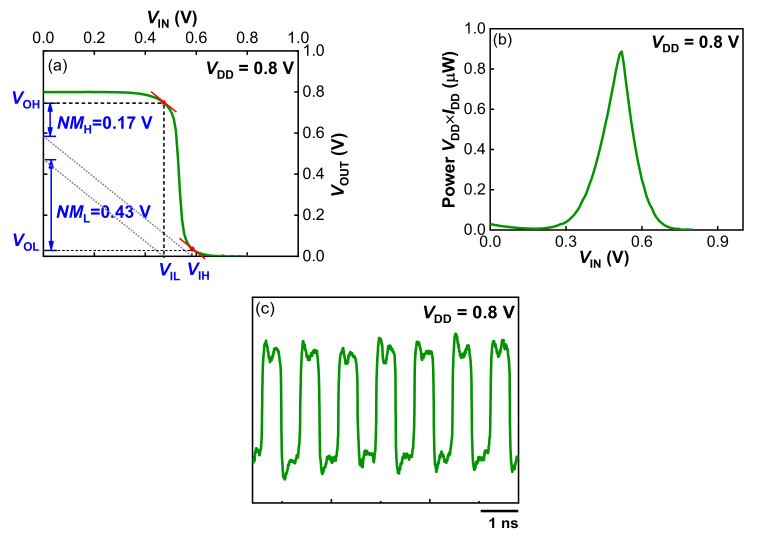
(**a**) Linear approximation of the VTC to estimate the static NMs, (**b**) Static power consumption with respect to *V*_IN_, (**c**) Characteristics of 101-stage CMOS RO based on low-temperature Schottky S/D FinFETs.

The CMOS inverter voltage transfer characteristics (VTC) at *V*_DD_ ranging from 0.3 V to 1 V by step of 0.1 V are presented in Figure 8a. The source of Schottky S/D NMOS was connected to the ground potential, while the source of Schottky S/D PMOS was attached to *V*_DD_. Both transistors shared the silicided drain contact forming the output terminal of inverter *V*_OUT_, as illustrated in Figure 1b. Well-behaved VTC was obtained, with a low-to-high output dynamic that reached rail-to-rail supply voltage range. This indicates that the subthreshold leakage currents of both transistors were sufficiently low to not degrade high and low logic states. It is noted that the transition of the inverter VTC was not located at *V*_DD_/2, due to the uncompensated asymmetry of *V*_TH_ between pFinFETs and nFinFETs (Figure 6). The transition of the inverter VTC was shifted by the same amount of about 0.14 V. A gate metal work function adjustment could be applied to optimize *V*_TH_ symmetry to further improve the inverter VTC. Almost a constant voltage gain (∆*V*_OUT_/∆*V*_IN_) of 18 *v/v* was achieved at *V*_DD_ in the range of 0.3 V ~ 0.8 V (Figure 8b), suggesting a great potential of our inverter in low-power and high-performance 3D sequential integration. In order to estimate the noise margin (*NM*), a piecewise approximation of the VTC was used here to determine the boundary of the transition zone. As illustrated in Figure 9a, the output voltage and input voltage for high (*V*_OH_, *V*_IH_) as well as for low (*V*_OL_, *V*_IL_) were defined by the position of points where d*V*_OUT_/d*V*_IN_ = −1. *NM* for high input (NM_H_ = V_OH_ − V_IH_) of 0.17 V and *NM* for low input (*NM*_L_ = *V*_IL_ − *V*_OL_) of 0.43 V at *V*_DD_ = 0.8 V were obtained. Figure 9b shows the static power consumption as a function of *V*_IN_ at *V*_DD_ = 0.8 V. The maximum static power consumption for *V*_IN_ sweeping from 0 V to 0.8 V at *V*_DD_ = 0.8 V was less than 0.9 μW. In Figure 9c, CMOS ring oscillators (RO) composed by 101 stages were successfully operated in low-temperature Schottky S/D FinFETs. Again, this validates the feasibility of Schottky S/D FinFETs as the top-tier devices in 3D sequential technology.

## 4. Conclusions

In conclusion, low-temperature complementary Schottky S/D FinFETs were proposed as the top-tier devices for 3D sequential integration and were experimentally demonstrated in this work. The thermal budget for fabrication was no more than 500 °C. and the entire process flow was fully compatible with current Si technology. With optimal SDE engineering and competitive *I*_ON_ values of 76.07 μA/μm and 48.57 μA/μm, *I*_ON_/*I*_OFF_ ratios of 7 × 10^5^ and 1 × 10^6^ at *V*_DD_ = 0.8 V were obtained for pFinFETs and nFinFETs, respectively. Excellent CMOS inverter and functional CMOS RO are successfully explored, offering a new method of high-performance 3D VLSI CMOS integration.

## Figures and Tables

**Table 1:** Split of thermal budget for SDE formation.

Item ^1^	310 °C SDE ^1st^	450 °C SDE ^last^	550 °C SDE ^last^
Silicidation annealing	310 °C 60 s	310 °C 60 s	500 °C 30 s
Drive-in annealing	NA	450 °C 60 s	550 °C 60 s

^1^ All annealing steps were performed by RTA.

## Data Availability

The data presented in this study are available on request from the corresponding author.
